# Mother-reliant or self-reliant: the germination strategy of seeds in a species-rich alpine meadow is associated with the existence of pericarps

**DOI:** 10.1093/aob/mcae086

**Published:** 2024-05-29

**Authors:** Xiao-Qing Li, Hong-Yu Zhu, Yong-Deng He, Anne Christine Ochola, La Qiong, Chun-Feng Yang

**Affiliations:** School of Ecology and Environment, Tibet University, Lhasa 850000, China; Key Laboratory of Biodiversity and Environmental on the Qinghai-Tibetan Plateau, Ministry of Education, School of Ecology and Environment, Tibet University, Lhasa 850000, China; State Key Laboratory of Plant Diversity and Specialty Crops, Wuhan Botanical Garden, Chinese Academy of Sciences, Wuhan 430074, China; State Key Laboratory of Plant Diversity and Specialty Crops, Wuhan Botanical Garden, Chinese Academy of Sciences, Wuhan 430074, China; College of Forestry, Jiangxi Agricultural University, Nanchang 330045, China; State Key Laboratory of Plant Diversity and Specialty Crops, Wuhan Botanical Garden, Chinese Academy of Sciences, Wuhan 430074, China; State Key Laboratory of Plant Diversity and Specialty Crops, Wuhan Botanical Garden, Chinese Academy of Sciences, Wuhan 430074, China; School of Ecology and Environment, Tibet University, Lhasa 850000, China; Key Laboratory of Biodiversity and Environmental on the Qinghai-Tibetan Plateau, Ministry of Education, School of Ecology and Environment, Tibet University, Lhasa 850000, China; State Key Laboratory of Plant Diversity and Specialty Crops, Wuhan Botanical Garden, Chinese Academy of Sciences, Wuhan 430074, China

**Keywords:** Flowering time, pericarp, seed mass, germination, maternal effect, phylogeny

## Abstract

**Background and Aims:**

Some plants germinate their seeds enclosed by a pericarp, whereas others lack the outer packaging. As a maternal tissue, the pericarp might impart seeds with different germination strategies. Plants in a community with different flowering times might separately disperse and germinate their seeds; therefore, flowering time can be considered as one manifestation of maternal effects on the offspring. The mass of the seed is another important factor influencing germination and represents the intrinsic resource of the seed that supports germination. Using seeds from a species-rich alpine meadow located in the Hengduan Mountains of China, a global biodiversity hotspot, we aimed to illustrate whether and how the type of seed (with or without a pericarp) modulates the interaction of flowering time and seed mass with germination.

**Methods:**

Seeds were germinated in generally favourable conditions, and the speed of germination [estimated by mean germination time (MGT)] was calculated. We quantified the maternal conditions by separation of flowering time for 67 species in the meadow, of which 31 produced seeds with pericarps and 36 yielded seeds without pericarps. We also weighed 100 seeds of each species to assess their mass.

**Key Results:**

The MGT varied between the two types of seeds. For seeds with pericarps, MGT was associated with flowering time but not with seed mass. Plants with earlier flowering times in the meadow exhibited more rapid seed germination. For seeds without a pericarp, the MGT depended on seed mass, with smaller seeds germinating more rapidly than larger seeds.

**Conclusions:**

The distinct responses of germination to flowering time and seed mass observed in seeds with and without a pericarp suggest that germination strategies might be mother-reliant for seeds protected by pericarps but self-reliant for those without such protection. This new finding improves our understanding of seed germination by integrating ecologically mediated maternal conditions and inherent genetic properties.

## INTRODUCTION

The seeds of angiosperms develop within the fruit. The specific fruit type of a plant species is of significant importance in seed dispersal, defence and subsequent growth of seedlings, linking the reproductive capacity of individual plants to the broader regeneration of the plant community (Wang *et al.*, 2022). Fruits can be classified into two main categories: dry and fleshy fruits. Dry fruits are divided into dehiscent and indehiscent types, based on whether the pericarp splits open after reaching maturity (Lorts and Briggeman, 2008). Seeds with pericarps are typically from indehiscent fruits, such as achenes (e.g. Asteraceae and Polygonaceae) and nutlets (e.g. Lamiaceae), whereas seeds without pericarps are always found in plants with dehiscent fruits, such as capsules (e.g. Scrophulariaceae), legumes and siliques (e.g. Brassicaceae). Seeds with and without pericarps can vary in their germination strategies owing to the presence or absence of a pericarp. For example, Vercellino *et al.* (2019) suggested that the existence of a pericarp could impede the germination process, indicating an insensitive germination response to environmental cues. For seeds with pericarps, additional maternal tissue can subject its germination strategy to maternal effects (Donohue, 2009). On the contrary, the germination of seeds without pericarps can be comparatively sensitive to environmental cues, and this sensitivity is closely related to seed mass (Fernández‐Pascual *et al.*, 2022). This suggests that the germination strategies of seeds with and without pericarps might be associated with maternal conditions and intrinsic resources. However, the interplay between maternal conditions, seed mass and seed type in the germination strategy remains unclear.

Seed germination is an essential developmental transition sensitive to environmental cues (D’Aguillo and Donohue, 2023). Mean germination time (MGT) is an indicator that estimates the speed of germination (Lozano‐Isla *et al.*, 2019), reflecting the germination strategy of seeds. It is crucial for individual fitness, influencing population dynamics, local persistence and species distribution within landscapes (Gremer *et al.*, 2020). Therefore, germination speed is an effective mechanism of habitat selection; it has been shown to influence the environment that subsequent life stages experience (Norden *et al.*, 2009; Chen *et al.*, 2018). Rapid germination can provide a competitive advantage with a relatively long growing season before reproduction, whereas slow germination might reduce the risk of exposure to adverse conditions (D’Aguillo and Donohue, 2023). Consequently, germination speed serves as a strategy to enhance long-term reproductive success by spreading risk over time (Venable, 2007).

Flowering timing represents a pivotal life-history event within plant phenology and is a crucial element within plant ecology (Wolkovich *et al.*, 2014). The interspecific variation of phenology is involved in niche differentiation and is therefore likely to play an imperative role in community structure and dynamics (Craine *et al.*, 2012). Flowering time is vital to achieving plant reproductive success and influences the expression of traits in offspring (Liu *et al.*, 2021). Flowering time is a property of the maternal parent and affects germination by determining the seed dispersal season (Ehrlén *et al.*, 2015; Galloway *et al.*, 2018). For example, studies have indicated that flowering time directly influences seed germination speed (Xu *et al.*, 2014; Zhang *et al.*, 2014*a*). This influence on the environments of the offspring and subsequent life-history events through the expression of maternal conditions is referred to as ‘maternal effects’ (Galloway and Burgess, 2009).

Seed germination is highly related to seed mass (Fernández‐Pascual *et al.*, 2019), reflecting many evolutionary forces (Moles *et al.*, 2005). Seed mass represents the intrinsic level of resources (Germain and Gilbert, 2014). Some studies suggest that larger seeds should germinate more quickly to escape the attraction of seed predators to their high resource content, thus reducing risk and increasing the chances of seedling establishment (Huang *et al.*, 2016). There are also studies proposing the opposite view, suggesting that smaller seeds germinate faster than larger seeds (Norden *et al.*, 2009). In alpine plants, it has been observed that seed mass is negatively correlated with germination speed (Wang *et al.*, 2021) or positively correlated with MGT (Zhang *et al.*, 2014*b*). The thicker seed coat of large seeds gives them more dormancy characteristics, while also slowing the entry of water and oxygen, resulting in their slow germination (Radchuk and Borisjuk, 2014). On the contrary, smaller seeds tend to be more sensitive to environmental cues (Liu *et al.*, 2013). It is necessary to explore the dependence of germination speed on seed mass for seeds both with and without pericarps in the same germination conditions.

In this study, our objective was to illustrate whether and how the type of seed (with and without a pericarp) modulates the interaction of flowering time and seed mass with germination speed. To account for the influence raised by different germination environments, an ideal study system is to incorporate many species that grow in a similar environment to perform germination experiments in generally optimized conditions. We collected seeds (>500 for each species) from 67 species in a species-rich alpine meadow in the Hengduan Mountains for germination experiments. We aimed experimentally to uncover the germination strategies (e.g. germination speed) of seeds with and without pericarps by revealing their patterns of dependency on maternal conditions (estimated by flowering time) or intrinsic resource levels (seed mass). This differentiation in germination strategies might contribute to niche segregation among diverse plants within a community.

## MATERIALS AND METHODS

### Study area

The alpine meadow is located on the eastern edge of the Hengduan Mountains (27°37ʹN, 99°47ʹE; 3360 m a.s.l.), China. It covers a valley basin of ~12 ha surrounded by mountains. The mean annual temperature is 6.3 °C, with monthly averages ranging from −2.3 °C in January to 13.9 °C in July. Annual precipitation averaged 651.1 mm between 1958 and 2020, with 81.3 % falling from June to September (Zhang *et al.*, 2023).

### Flowering time of species in the meadow

More than 100 species of flowering plants grow in this meadow, and the flowering season typically extends from late May to early September. During the 2020 flowering season, a total of 299 plots (2 m × 2 m) were established, evenly distributed throughout the study area, covered with flowering plants. We conducted surveys every 7–10 days (depending on weather conditions), for a total of nine times. We recorded fully open flowers for each species on each survey day. For some species of monospermic fruits (e.g. Asteraceae and Polygonaceae) and cremocarp (Apiaceae), we calculated the number of flowers using inflorescence as a unit. We defined the peak flowering date of each species as the survey day on which the number of flowers was highest (Wolf *et al.*, 2017). The flowering time of each species was established as the number of days between the peak flowering date and 1 June (Bolmgren and Eriksson, 2015).

### Seed collection, storage, type and mass

We collected the ripened fruits before their natural dispersal. Seeds were classified into two groups according to whether the pericarp ruptured when the fruit reached full ripeness for dispersal. Finally, we collected seeds from 67 species belonging to 19 families, including 31 species for seeds with pericarps and 36 species for seeds without pericarps. The quantity of seeds for each species was >500 to meet the demand for further analyses and experiments. In our study, seeds with pericarps are from indehiscent fruits, such as achenes, nutlets and cremocarps, whereas seeds without pericarps are from dehiscent fruits, such as capsules, legumes, siliques and follicles. We dried these fruits in room conditions (~20 °C and 50 % relative humidity) for 3 weeks and removed the seeds from the cracked fruits. We randomly selected 100 seeds and weighed them to the nearest 0.01 mg, performing three replicates for each species, to determine the seed mass. All dried seeds were stored in a refrigerator at 4 °C for ~6 months until germination experiments. Dry and cold storage of seeds can induce after-ripening (Peng *et al.*, 2021). Meanwhile, it can also maintain the dormancy of unburied seeds that remain unaffected (Holdsworth *et al.*, 2008; Peng *et al.*, 2021). Therefore, this storage treatment allows us to detect differences in dormancy levels among different species when germinating in favourable conditions.

### Germination experiment

Based on the information provided by the Germplasm Bank of Wild Species, the majority of the plants gathered from regions adjacent to our research site can germinate better when the photoperiod is 12 h and the temperature is 25 °C in the daytime and 15 °C at night, respectively. We adopted these measurements to create favourable conditions for our germination experiment. The seeds were sterilized with 5 % NaOCl and washed with distilled water prior to germination. Three replicates of 40 intact seeds were incubated on moist filter papers in 9-cm-diameter Petri dishes for each species. Germination experiments were conducted in temperature- and light-controlled incubators (HP300GS-C, China) for 30 days. Each day, germinated seeds (radicle protruding from the seed coat) were recorded and removed from the Petri dishes, and decaying seeds were also removed (Wang *et al.*, 2021). Distilled water was added to the filter paper as needed.

### Germination response variables

The MGT represents the average duration for a certain proportion of seeds to germinate in relationship to the total number of seeds germinated at the time of evaluation, which is the time it takes for half the seeds to germinate, indicating the speed of germination. We calculated the MGT with the package ‘GerminaR’ in R (Lozano‐Isla *et al.*, 2019). Additionally, we also calculated the final germination proportion, which represents the proportion of seeds that successfully complete the germination process. It was determined by dividing the number of germinated seeds by the total number of viable seeds (Wang *et al.*, 2021).

### Statistical analyses

In this study, statistical models were used in a phylogenetic controlled framework, because germination between species might display a phylogenetic conservatism. Initially, we constructed a phylogenetic tree for all species studied, which was extracted from a previously published mega-tree using the ‘phylo.maker’ function in the R package ‘V.PhyloMaker’ (Jin and Qian, 2019), based on the classification of flowering plants (Smith and Brown, 2018). The limitation of this approach is that the binary structure of a mega-tree cannot be established for species within a genus, because they share similar nodes. Therefore, we reconstructed a phylogenetic tree of our study species based on genetic markers. A DNA supermatrix was generated from two chloroplast sequence regions (*rbcL* and *matK*) and the nuclear ribosomal internal transcribed spacer (ITS). The ITS regions were aligned within families, then concatenated to the *rbcL* and *matK* alignments. The DNA supermatrix was then analysed using RAxML via the CIPRES supercomputer cluster to infer a maximum likelihood (ML) phylogeny using the APG IV phylogenetic tree (obtained from the mega-tree) as a constraint. A constraint tree approach helps to ensure that the basal topology of a molecular community phylogeny is consistent with the global working hypothesis for the basal topology of angiosperms (Yang *et al.*, 2014).

We used phylogenetic generalized least squares (PGLS) models to test the effect of flowering time, seed mass and seed types on MGT. This method integrates relatedness information (provided as a phylogenetic tree) into the error term of a generalized least-squares (GLS) model, while assuming a specific model of trait evolution. PGLS allows the incorporation of an anticipated model of evolution and phylogeny into the variance–covariance matrix, thus addressing the lack of independence among species (Gaoue *et al.*, 2021). PGLS was implemented using the ‘gls’ function from the ‘nlme’ R package (Pinheiro *et al.*, 2020), and the correlation argument was set via the ‘corBrownian’ function from the ‘ape’ R package (Paradis and Schliep, 2019), with the ‘gamma’ parameter set to one. Initially, we used PGLS models to examine the influence of flowering time, seed mass and seed types on MGT, and whether the association between MGT and flowering time or seed mass varied between seeds with and without pericarps (i.e. MGT ~ flowering time + seed mass + seed types + flowering time: seed types + seed mass: seed types). We also used PGLS regression separately to analyse the influence of flowering time and seed mass on MGT between species for seeds with and without pericarps, respectively (i.e. MGT ~ flowering time + seed mass + flowering time: seed mass for groups of seeds with and without pericarps, respectively).

We also tested the effect of flowering time and seed mass on the final germination proportion by fitting a Bayesian logistic phylogenetically informed generalized mixed model using the ‘MCMCglmm’ R package (Hadfield, 2010). The model used weakly informative priors and was run for 50 000 iterations with a burn-in of 1000 and a thinning interval of 100, generating 90 posterior distributions. Mean parameter estimates and 95 % credible intervals (CI) were calculated. Parameters with CIs overlapping zero were considered non-significant.

In all models, the seed mass was log_10_-transformed. All statistical analyses were performed using the program R v.4.1.3 (R Core Team, 2023).

## RESULTS

A considerable disparity of 635 times in seed mass was observed among species, ranging from 1.83 mg in *Saxifraga pallida* to 1163.90 mg in *Iris bulleyana*. The mean value and the range for the final germination proportion and MGT were 42.54 % (0 %–100 %) and 13.40 days (0–27.5 days), respectively. The final germination proportion was <50 % in 40 species and >50 % in 27 species; moreover, it was not influenced by either flowering time (MCMCglmm, posterior mean = 0.010; CI = −0.014 to 0.045; *P* = 0.478) or seed mass (posterior mean = −0.756; CI = −1.687 to 0.250; *P* = 0.155).

**Fig. 1.**
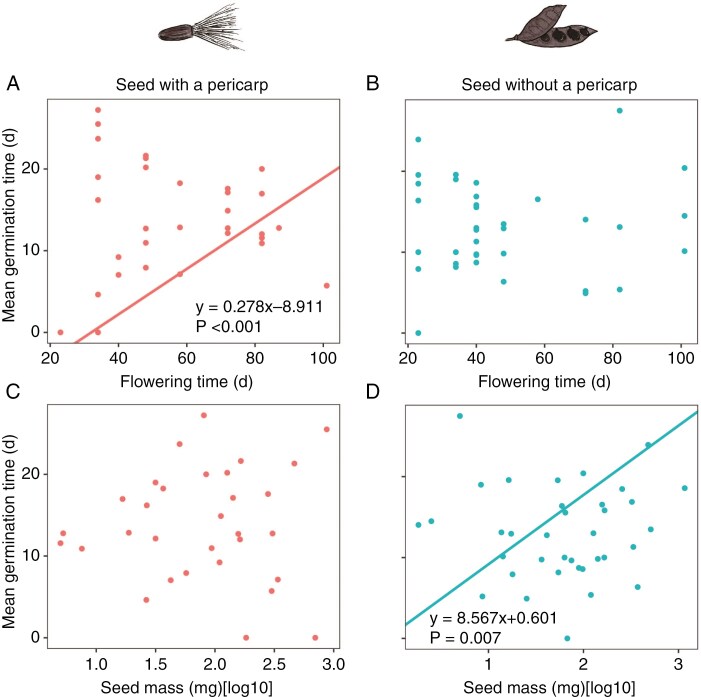
The mean germination time (in days) in relationship to flowering time (in days; a, b) and seed mass (in milligrams; c, d) of seeds with a pericarp (red) and seeds without a pericarp (blue) under phylogenetic generalized least-squares regressions. For details of the statistical results, see Table 2.

Our results indicated that MGT differed slightly between the two seed types (*P* = 0.062; Table 1). Furthermore, flowering time significantly affected MGT (*P *< 0.001; Table 1) and displayed an interactive effect with seed type to influence MGT (*P *= 0.003; Table 1). Additionally, MGT was significantly influenced by seed mass (*P *= 0.047; Table 1) but not by an interactive effect between seed mass and seed types (Table 1). Separate models revealed that for seeds with pericarps, MGT was positively influenced by flowering time (*P *< 0.001; Fig. 1A; Table 2) but not by seed mass (Fig. 1C; Table 2). On the contrary, for seeds without pericarps, MGT had a positive relationship with seed mass (*P *= 0.007; Fig. 1D; Table 2) but not with flowering time (Fig. 1B; Table 2).

**Table 1.** Statistics for the phylogenetic generalized least-squares model of the effect of flowering time, seed mass and seed types on the mean germination time. Significant effects are in bold (*P* < 0.05).

Explanatory variable	The mean germination time	
	*F*	*P*-value
Flowering time (FT)	12.491	**<0.001**
Seed mass (SM)	4.110	**0.047**
Seed type (ST)	3.608	0.062
FT: ST	9.554	**0.003**
SM: ST	0.509	0.478

**Table 2.** Statistics for the phylogenetic generalized least-squares model of the effect of flowering time and seed mass on the mean germination time of seeds with and without pericarps, respectively. Significant effects are in bold (*P* < 0.05).

Explanatory variable	Mean germination time of seeds with pericarps		Mean germination time of seeds without pericarps	
	*F*	*P*-value	*F*	*P*-value
Flowering time (FT)	20.901	**<0.001**	0.187	0.641
Seed mass (SM)	0.167	0.686	8.019	**0.007**
FT: SM	0.290	0.595	8.208	**0.007**

## DISCUSSION

Our experiments showed that the final germination proportion of plants in this alpine meadow was relatively low in favourable conditions (<50 % for the majority of species studied), indicating seed dormancy. The dormancy of alpine seeds helps to prevent germination immediately after dispersal, mitigating the risk of freezing (Tudela‐Isanta *et al.*, 2018). This suggests that our dry and cold storage before germination treatments does not break seed dormancy (Finch‐Savage and Leubner‐Metzger, 2006; Liu *et al.*, 2011), allowing us to detect that, for those seeds varying in their dormancy extents, whether and how germination strategies of seeds with and without pericarps were influenced by maternal condition (quantified by flowering time) and intrinsic resource levels (seed mass). Our research indicated that flowering time and seed mass have a significant impact on MGT, but not on the final germination proportion. Seeds with and without pericarps differed in MGT. Furthermore, the influence of seed mass and flowering time on MGT varied depending on the seed type. The flowering time influenced the MGT of the seeds with pericarps, suggesting a maternal effect. For seeds without pericarps, it was seed mass, an indicator of the level of seed intrinsic resources, that influenced MGT. This finding suggests that the seed type might play a pivotal role in determining germination strategies and thus provides new insights into alpine seed ecology by revealing how ecologically mediated maternal conditions and genetic properties are connected.

Our results indicated that, for seeds protected by pericarps, earlier-flowering plants exhibit a shorter germination time, indicating faster germination in comparison to late-flowering plants. Flowering time has been found to influence seed germination speed significantly, e.g. in *Campanula americana* (Galloway, 2002) and *Actaea spicata* (Ehrlén *et al.*, 2015). These findings that early-flowering plants tend to germinate rapidly at the intraspecific level were confirmed by our present results at the community level. This suggests that for seeds with pericarps, the safest method to initiate seed germination is to follow the growth conditions of their maternal plant. Additionally, the early-dispersal seeds produced by early-flowering plants can benefit from abundant resources and less competition; therefore, they germinate quickly in warm and extended sunlight (Satyanti *et al.*, 2019). The seeds of autumn-flowering plants are usually dispersed in late autumn and can have slow germination over winter or delayed germination in low-temperature and short-daylength conditions (Mondoni *et al.*, 2012; Tudela‐Isanta *et al.*, 2018). Our study infers that for seeds with pericarps, the maternal effects might transfer to seeds in terms of their germination strategies.

Seeds lacking pericarps are often dehiscent fruits, which detach from the pericarp and disperse from the maternal site. Our results indicated a significant positive correlation between MGT and seed mass for seeds without a pericarp. This suggests that, in the absence of pericarps, the initiation of seed germination is largely dependent on the stored resources of the seed, demonstrating a self-reliant strategy. Seeds without pericarps might be more sensitive to environmental cues than those with pericarps. This inference might explain the findings that, for seeds without pericarps, smaller seeds germinate in a shorter time, because they are more sensitive to changes in water and temperature (Rubio de Casas *et al.*, 2017). Additionally, smaller seeds have a wider dispersal range and rely more on their own resources for survival when a new environment is distant from the maternal plant (Donohue, 2005). Our present study confirms this and also suggests that the relatively high sensitivity of germination for small seeds might be manifested more in seeds without pericarps than in those protected by pericarps.

In conclusion, our study revealed that seeds with and without pericarps adopt distinct germination strategies. The germination speed for seeds with pericarps depends on the flowering time, an estimator of maternal condition, whereas for those without pericarps it is associated with their own resource level, the seed mass. The results enrich our understanding of seed ecology by incorporating ecologically mediated maternal conditions and inherent genetical properties. The more protection a seed receives, the more germination is associated with the conditions of mother plants. Conducting studies that include ecological and physiological factors related to the dispersal and defence mechanisms of seeds, both with and without pericarps, on a broad geographical scale is crucial for comprehending the difference in their germination strategy.

## Data Availability

Data are available from Figshare: https://doi.org/10.6084/m9.figshare.24151848
